# Supplementary material for: Evolutionary loss of foot muscle during development with characteristics of atrophy and no evidence of cell death
Source: eLife. 2019 Oct 15;8:e50645. doi: 10.7554/eLife.50645 (PMC6855805; doi:10.7554/eLife.50645)
Supplement: Figure 5—source data 1. — ‘Good’ represents striated localization of each protein to the sarcomere, and ‘bad’ refers to no distinguishable banded pattern of protein expression. In group 1, we saw myofibers with all three proteins properly localized suggesting disorganization follows an initial state of proper localization. When we compared the dRM and Drm categories, we saw loss of Desmin localization when Tropomyosin and Myosin were ‘good’ and almost no myofibers where Desmin was good and the others were bad. This suggests that Desmin is disorganized prior to Tropomyosin and Myosin. In group 2a, there were cells in the rmT category and almost none in the RMt category, suggesting Tropomyosin and Myosin are disorganized prior to Titin. Group 2b illustrates that both categories RmT and rMT appeared at similar frequency, suggesting it is unclear whether Tropomyosin or Myosin become disorganized prior to the other. In group 3, there were cells in the mtA category and none in the MTa category, suggesting Titin becomes disorganized before Alpha-actinin. Similarly, in group 4, there were cells in the mtY category but not in the MTy category suggesting Titin becomes disorganized before Myomesin. Due to shared antibody isotype for Alpha-actinin and Myomesin, the order of disorganization between these two could not be discerned. See Figure 5—source data 2–5 for full details of the percentage of myofibers in each category for each combination of multicolor immunofluorescence. [file elife-50645-fig5-data1.pdf]

Key
D = Good Desmin
d = Bad Desmin
R = Good Tropomyosin
r = Bad Tropomyosin
M = Good Myosin
m = Bad Myosin
T = Good Titin
t = Bad Titin
A = Good α-actinin
a = Bad α-actinin
Y = Good Myomesin
y = Bad Myomesin

		Jerboa foot muscles		
		**P0**	**P2**	**P4**
Group 1	**DRM**	63% ± 26%	23% ± 26%	17% ± 1%
	**dRM**	10% ± 2%	5% ± 6%	31% ± 6%
	**Drm**	0	2% ± 3%	0
Group 2a	**rmT**	8% ± 7%	7% ± 7%	23% ± 9%
	**RMt**	1% ± 3%	0	0
Group 2b	**RmT**	4% ± 2%	1% ± 3%	17%16%
	**rMT**	15% ± 18%	26% ± 11%	12% ± 15%
Group 3	**mtA**	0	25% ± 21%	25% ± 18%
	**MTa**	0	0	0
Group 4	**mtY**	5% ± 4%	36% ± 18%	27% ± 16%
	**MTy**	0	0	0
