# Supplementary material for: Evolutionary loss of foot muscle during development with characteristics of atrophy and no evidence of cell death
Source: eLife. 2019 Oct 15;8:e50645. doi: 10.7554/eLife.50645 (PMC6855805; doi:10.7554/eLife.50645)
Supplement: Figure 5—source data 2. — For the combination of Desmin, Tropomyosin and Myosin: P0 (two animals) hand n = 78 myofibers and foot n = 113 myofibers; P2 (two animals) hand n = 56 and foot n = 54 myofibers; P4 (two animals) hand n = 69 and foot n = 117 myofibers. For the combination of Desmin, Myosin, and Titin: P0 (two animals) hand n = 48 and foot n = 96 myofibers; P2 (two animals) hand n = 71 and foot n = 77 myofibers; P4 (two animals) hand n = 63 and foot n = 92 myofibers. [file elife-50645-fig5-data2.pdf]

Key	Stain: Desmin, Tropomyosin, Myosin						
D = Good Desmin<br>d = Bad Desmin<br>R = Good Tropomyosin<br>r = Bad Tropomyosin<br>M = Good Myosin<br>m = Bad Myosin		**Hand muscles**			**Foot muscles**		
		**P0**	**P2**	**P4**	**P0**	**P2**	**P4**
	**DRM**	94% ± 1%	87% ± 2%	96% ± 1%	63% ± 26%	23% ± 26%	17% ± 1%
	**drm**	4% ± 2%	5% ± 7%	0	16% ± 14%	48% ± 15%	36% ± 4%
	**dRM**	0	4% ± 5%	4% ± 1%	10% ± 2%	5% ± 6%	31% ± 6%
	**drM**	2% ± 3%	0	0	9% ± 13%	7% ± 10%	7% ± 9%
	**dRm**	0	4% ± 5%	0	2% ± 0%	6% ± 9%	9% ± 13%
	**Drm**	0	0	0	0	2% ± 3%	0
	**DrM**	0	0	0	1% ± 0%	5% ± 6%	0
	**DRm**	0	0	0	0	5% ± 7%	0

Key	Stain: Desmin, Myosin, Titin						
D = Good Desmin<br>d = Bad Desmin<br>M = Good Myosin<br>m = Bad Myosin<br>T = Good Titin<br>t = Bad Titin		**Hand muscles**			**Foot muscles**		
		**P0**	**P2**	**P4**	**P0**	**P2**	**P4**
	**DMT**	90% ± 14%	82% ± 13%	95% ± 4%	32% ± 9%	11% ± 15%	7% ± 5%
	**dmt**	0	7% ± 6%	2% ± 3%	26% ± 13%	47% ± 10%	21% ± 23%
	**dMT**	2% ± 2%	7% ± 4%	3% ± 1%	33% ± 7%	33% ± 23%	33% ± 18%
	**dmT**	8% ± 11%	0	0	10% ± 3%	2% ± 3%	38% ± 1%
	**dMt**	0	0	0	0	6% ± 8%	0
	**Dmt**	0	0	0	0	0	0
	**DmT**	0	1% ± 2%	0	0	0	0
	**DMt**	0	3% ± 4%	0	0	1% ± 2%	0
