# Supplementary material for: Evolutionary loss of foot muscle during development with characteristics of atrophy and no evidence of cell death
Source: eLife. 2019 Oct 15;8:e50645. doi: 10.7554/eLife.50645 (PMC6855805; doi:10.7554/eLife.50645)
Supplement: Figure 5—source data 3. — P0 hand (three animals; n = 125 myofibers) and foot (four animals; n = 225 myofibers); P2 (four animals) hand n = 118 and foot n = 183 myofibers; P4 (three animals) hand n = 104 and foot n = 172 myofibers. [file elife-50645-fig5-data3.pdf]

Key	Stain: Tropomyosin, Myosin, Titin						
R = Good Tropomyosin		**Hand muscles**			**Foot muscles**		
r = Bad Tropomyosin		**P0**	**P2**	**P4**	**P0**	**P2**	**P4**
M = Good Myosin	**RMT**	66% ± 30%	72% ± 25%	91% ± 7%	45% ± 33%	15% ± 21%	24% ± 17%
m = Bad Myosin	**rmt**	6% ± 4%	5% ± 5%	2% ± 4%	18% ± 13%	48% ± 12%	24% ± 5%
T = Good Titin	**rMT**	14% ± 25%	23% ± 27%	3% ± 5%	20% ± 19%	24% ± 11%	12% ± 15%
t = Bad Titin	**rmT**	0	0	0	8% ± 7%	7% ± 7%	23% ± 9%
	**rMt**	4% ± 5%	0	0	1% ± 2%	5% ± 9%	0
	**Rmt**	3% ± 2%	0	0	2% ± 4%	0	0
	**RmT**	5% ± 9%	0	4% ± 4%	3% ± 3%	2% ± 4%	17% ± 16%
	**RMt**	2% ± 4%	0	0	1% ± 3%	0	0
