# Supplementary material for: Evolutionary loss of foot muscle during development with characteristics of atrophy and no evidence of cell death
Source: eLife. 2019 Oct 15;8:e50645. doi: 10.7554/eLife.50645 (PMC6855805; doi:10.7554/eLife.50645)
Supplement: Figure 5—source data 4. — P0 hand (three animals) n = 156 and foot n = 182 myofibers; P2 (four animals) hand n = 189 and foot n = 203 myofibers; P4 hand (three animals; n = 104 myofibers) and foot (four animals; n = 172 myofibers). [file elife-50645-fig5-data4.pdf]

Key	Stain: Myosin, Titin, α-actinin						
M = Good Myosin<br>m = Bad Myosin<br>T = Good Titin<br>t = Bad Titin<br>A = Good α-actinin<br>a = Bad α-actinin		**Hand muscles**			**Foot muscles**		
		**P0**	**P2**	**P4**	**P0**	**P2**	**P4**
	**MTA**	76% ± 1%	84% ± 17%	93% ± 6%	55% ± 23%	27% ± 28%	17% ± 3%
	**mta**	10% ± 5%	9% ± 13%	2% ± 4%	17% ± 9%	38% ± 32%	34% ± 12%
	**mTA**	12% ± 6%	3% ± 4%	3% ± 3%	12% ± 6%	6% ± 8%	23% ± 15%
	**mtA**	1% ± 1%	3% ± 3%	2% ± 3%	0	25% ± 21%	25% ± 18%
	**mTa**	0	0	0	0	0	0
	**Mta**	0	0	0	0	0	0
	**MtA**	1% ± 1%	1% ± 2%	0	16% ± 13%	4% ± 3%	1% ± 2%
	**MTa**	0	0	0	0	0	0
