# Supplementary material for: Evolutionary loss of foot muscle during development with characteristics of atrophy and no evidence of cell death
Source: eLife. 2019 Oct 15;8:e50645. doi: 10.7554/eLife.50645 (PMC6855805; doi:10.7554/eLife.50645)
Supplement: Figure 5—source data 5. — P0 (three animals) hand n = 130 and foot n = 159 myofibers; P2 (three animals) hand n = 126 and foot n = 142 myofibers; P4 hand (three animals; n = 98 myofibers) and foot (four animals; n = 164 myofibers). [file elife-50645-fig5-data5.pdf]

**Key**	**Stain: Myosin, Titin, Myomesin**						
M = Good Myosin		**Hand muscles**			**Foot muscles**		
m = Bad Myosin		**P0**	**P2**	**P4**	**P0**	**P2**	**P4**
T = Good Titin	**MTY**	100%	82% ± 9%	95% ± 9%	83% ± 7%	29% ± 12%	11% ± 10%
t = Bad Titin	**mty**	0	3% ± 1%	2% ± 4%	3% ± 5%	19% ± 10%	22% ± 9%
Y = Good Myomesin	**mTY**	0	10% ± 12%	2% ± 4%	9% ± 5%	13% ± 19%	24% ± 27%
y = Bad Myomesin	**mtY**	0	3% ± 6%	1% ± 2%	5% ± 4%	36% ± 18%	27% ± 16%
	**mTy**	0	0	0	0	0	0
	**Mty**	0	0	0	0	0	0
	**MtY**	0	1% ± 2%	0	0	4% ± 6%	16% ± 22%
	**MTy**	0	0	0	0	0	0
